# Effects of caloric and time restriction diets on kidney health in rat model of postmenopausal acute kidney injury: An apoptosis and histopathological study 

**DOI:** 10.22038/IJBMS.2022.61512.13609

**Published:** 2022-03

**Authors:** Fatemeh Darvishzadeh Mahani, Mohammad Khaksari, Maryam Iranpour, Zahra Soltani, Alireza Raji-Amirhasani, Zahra Hajializadeh

**Affiliations:** 1 Endocrinology and Metabolism Research Center, Kerman University of Medical Sciences, Kerman, Iran; 2 Department of pathology, Afzalipour Faculty of Medicine, Kerman University of Medical Sciences, Kerman, Iran; 3 Pathology and Stem Cells Research Center, Kerman University of Medical Sciences, Kerman, Iran; 4 Physiology Research Center, Institute of Neuropharmacology, Kerman University of Medical Sciences, Kerman, Iran

**Keywords:** Acute kidney Injury, Apoptosis, Calorie restriction, Diet, Histopathological findings, Time restriction

## Abstract

**Objective(s):**

Lifestyle and eating habits affect the health and function of the body’s organs, including the kidneys. The current study was carried out to determine the effects of two types of diet programs, including time restriction (TR) and calorie restriction (CR) on the histopathological changes and apoptotic molecules during acute kidney injury (AKI) in postmenopausal rats.

**Materials and Methods:**

In this study the female rats were divided into two groups of ovariectomized (OVX) and ovary-intact (sham), then they were placed on TR and CR diets for 8 weeks; afterward, AKI was induced by injection of glycerol. Functional indices, histopathological changes, Bax, and Bcl2 were measured before and after AKI.

**Results:**

After AKI, creatinine, serum urea, urinary albumin excretion, kidney tissue Bax, and Bax/Bcl2 ratio increased, while glomerular ﬁltration rate (GFR) and kidney tissue Bcl2 decreased compared with before AKI. Histopathological indices (inflammation, cellular necrosis, cell vacuolization, tubular dilatation, intratubular cast, and congestion) also confirmed renal injury. TR and CR diets improved renal injury indices and prevented an increase in the Bax/Bcl2 ratio. However, in some indices, the effects of two diets on OVX animals were not observed. In addition, none of the diets could decrease kidney weight/body weight ratio (KW/BW). The histopathological finding also showed improvement of renal status in both groups, especially in the CR diet.

**Conclusion:**

The results indicated that TR and CR diets had renoprotective effects against AKI by reducing the Bax/Bcl2 ratio and improving apoptosis. The effects of CR were more than TR.

## Introduction

An acute kidney failure is defined as acute kidney injury (AKI), resulting in an increase in creatinine, serum urea, abrupt decrease in the glomerular ﬁltration rate (GFR) (1) and/or increased urinary excretion (2). AKI is one of the most common complications in hospitalized patients, especially in the elderly. Despite the clinical importance of AKI, no perfect prevention or treatment has yet been found, and AKI is also associated with increased morbidity and mortality (3). AKI means loss of renal structural integrity, leading to renal failure (4). A number of pathologic processes cause AKI, including changes in local microvascular blood ﬂow, intratubular obstruction, immunological and inﬂammatory processes, and endothelial and epithelial cell death (5). Previous studies have shown that changes in kidney structure depend on the severity and type of the injury. For instance, there is tubular injury in patients with septic AKI (4). The ﬁndings showed a correlation between clinical deﬁnitions of AKI grade and pathologic kidney injury (6). The AKI mortality rate of men is twice that of women, and male rats have been reported to be more susceptible to injury than females. However, this protection decreases with age and menopause, suggesting that estradiol is a renoprotective factor (7). It has been shown that estrogen deﬁciency can accelerate glomerulosclerosis and kidney dysfunction in mice (8), and estradiol therapy after ovariectomy has been demonstrated to normalize the diminished renal function (9).

Several studies have indicated that dietary intervention is beneficial for different functions of organs. Among the types of dietary interventions, two diet programs can be mentioned, including time restriction (TR), which limits daily food consumption to 4–12 hr, and calorie restriction (CR), which reduces daily calorie intake by 15–40%. There is evidence that reducing daily calorie intake and periodic fasting can prevent diseases and extend longevity (10). For many years, mild dietary restriction has led to beneficial multisystemic effects and activated cellular stress response pathways to protect cells and tissues from injury (11).

CR and fasting have been shown to protect organs such as the kidneys from ischemic attacks (12). It has been proven that CR can increase longevity and improve general and metabolic health in all species (13). Mitchell *et al*. (2018) showed that AKI was prevented in renal ischemia-reperfusion injury (IRI) by a reduction in daily calorie intake by 30% over short time periods (14). CR is an attractive strategy for organ protection, and is one of the strongest preconditioning protocols, increasing stress resistance (15). Fasting or TR is a powerful nutritional intervention to reverse the numerous pathophysiological mechanisms involved in tissue injury and fibrosis after renal IRI (16). The renoprotective effects on fasting have been proven in early AKI. In other words, several studies have shown that short-term dietary interventions reduce tubular damage, cell death, inflammation, oxidative stress, and mitochondrial dysfunction after AKI (17). 

 A process of programmed cell death is called apoptosis. It is characterized by reduction of volume, cell surface blebbing, chromatin condensation, DNA internucleosomal cleavage, and apoptotic bodies formation (18). It has been believed that renal dysfunction is caused by apoptosis (19). Proximal tubule epithelial cells are very sensitive to apoptosis, and injury at this site can lead to organ failure. Apoptosis can be caused by several common renal injuries in the kidney, such as ureteral obstruction, radiation, toxic injury, and ischemia (5). Moreover, in glycerol-induced renal injury, apoptosis has been observed through the mitochondrial pathway and an increase in Bax level and a decrease in Bcl-2 level. (20). It has also been shown that apoptotic activity in the elderly kidney is reversed as a result of the antioxidant and anti-aging actions of CR (21). 

Considering the effective role of estrogen in impeding AKI and also the positive effects of TR and CR diets in preventing diseases, the objectives of the present study in postmenopausal model of rats included determining the effects of the diets mentioned above on the severity of glycerol induced AKI through histopathological indices, as well as determining the role of Bax and Bcl2 molecules involved in apoptosis after AKI. 

## Materials and Methods

***Animals***

The current study was carried out on 72 adult female Wistar rats (180–200 g). The rats were kept under natural conditions of 12:12 hr light-dark cycle at 24 °C in the animal center of Afzalipour School of Medicine. All tests were performed based on ethical instructions of the Institutional Animal Care Committee of Kerman University of Medical Sciences (ethics code: IR.KMU.REC.1398.469) and the national guidelines for the care and use of laboratory animals.

The animals were divided into two groups of ovary-intact (sham) and ovariectomized (OVX) rats; each group received the standard (SD), CR, and TR diets for 2 months. Then, the groups were divided into two subgroups of A and B. In subgroup A, kidney injury indices and biochemical parameters of kidney tissue and serum were measured, then the rats were sacrificed. In subgroup B, at the end of the second month, AKI was induced, and the factors mentioned above and histopathological indicators were measured after AKI (22). There were six rats in each subgroup.

***Sham groups***

*Control (Sham CTL)*

Ovary-intact rats in this group received SD (Pars animal, Iran), which contained 5.7%, 22.1%, and 72.2% calories from fat, protein, and carbohydrates, respectively (the total amount of calorie was 3.1 kc/g)(23). The rats were divided into two subgroups of A and B by the end of the second month.

*Time restriction (Sham TR)*

 This group was also similar to the control group; however, they had only 8 hr of daily free access to food (23).

*Calorie restriction (Sham CR)*

This group was similar to the control group; however, it received only 70% of the control group’s food (10).

***OVX groups***

**Figure 1:**
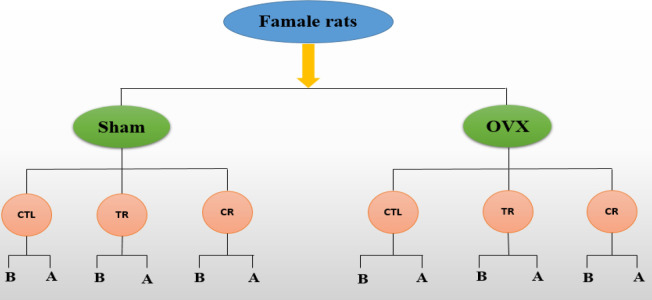
Schematic diagram of the classification of the animals. B: blood, U: urine, T: tissue, SD: standard diet, TR: time restriction diet, CR: calorie restriction diet, OVX: ovariectomized, Sham: ovary-intact, and CTL: control

 The rats were classified in the same way as the sham groups (OVX CLT, OVX TR, and OVX CR); however, they were ovariectomized (Figure1).

***Drugs***

Xylazine and ketamine were purchased from Alfasan Company (Utrecht, the Netherlands) and glycerol from Kimia Gostar Pooyesh Company (Iran).

***Bilateral ovariectomy***

A small longitudinal incision was made in the abdomen after anesthesia by xylazine and ketamine (80/10 mg/kg intraperitoneally)(24, 25). Ovaries were identified and removed after opening skin, fascia, and abdominal muscles; then, 1–2 ml of saline solution was poured into the abdomen, and the skin and muscles were sutured. A similar incision was made in the sham surgery, but the ovaries were not removed. The subsequent tests were performed 2 weeks after ovariectomy (26, 27).

***Meal size calculation and use of CR***

The amount of food intake of one week was measured in the CTL group to determine the meal size in the CR group, in which the animals had free access to the food, and the amount of daily intake was calculated as an average. Then, 70% of the daily intake amount in the group with free access to food was measured and provided for the CR group for 2 months (28).

***AKI induction***

Rats were deprived of water for 24 hr at the end of the 2 months to induce AKI, and then hypertonic glycerol solution (50% dissolved in sterile saline) was prepared and a single dose (10 ml/kg, im) of this solution was equally injected into both lower limbs of the rat. Twenty-four hours after the glycerol injection, nephropathy developed rapidly. Hypertonic glycerol causes rhabdomyolysis, which in turn causes myoglobinuria and leads to the development of nephrotoxicity and ischemia in the kidney. In the current study, creatinine, serum urea, albuminuria, and GFR were measured to confirm AKI induction (29).

***Bodyweight and kidney weight measurement***

A digital scale was used to measure the animal weights at the beginning and the end of the study. Furthermore, the kidneys of all animals were removed after sacrificing them, the surrounding adipose tissue was cleaned, and weighed by a digital scale.

***Measurement of urea and creatinine levels and BUN/CR ratio***

 Blood samples were taken from the choroid sinus and were immediately centrifuged, and the serum was separated one day before and one day after AKI induction to measure the indicators in serum. Furthermore, one day before and one day after AKI induction, urine was collected by a metabolic cage to measure the indicators in urine (Behboud Tahghigh, Kerman, Iran). The ELISA test was used to measure the urea and creatinine levels in serum and urine according to the kit instructions (MAN, Iran). The values were reported in mg/dl (30).

***Measurement of 24-hr urinary albumin excretion***

Using a metabolic cage, 24-hr urine of the animals was collected before and 24 hr after AKI induction, and the ELISA test was used to measure urinary albumin concentration according to the kit instructions (MAN, Iran). Afterward, the urinary albumin excretion (albuminuria) levels in 24 hr were measured using a 24-hr urine volume according to the following equation, and the values were reported in mg/24hr (31).

Albumin excretion in 24 hr = (urine volume in 24 hr × concentration of urine albumin)

***GFR measurement***

Rats were kept in a metabolic cage, and their urine was collected. This was performed before and 1 day after the AKI induction. Then, based on the 24-hr volume of urine and creatinine concentration in urine and serum, GFR (ml/24hr) was measured based on creatinine clearance according to the following equation (32): 

GFR=urine volume in 24 hr×concentration of urine creatinineconcentration of serum creatinine

Finally, GFR calculations were corrected for body weight (BW) and expressed as ml/24hr/100g bw.

***Histopathological procedures***

The left kidney was fixed in 10% neutral formalin solution and embedded in paraffin. Hematoxylin and eosin technique was used for staining tissue sections. A pathologist (blind to the study) was asked to determine the kidney tissue damage score (KTDS). The samples were scored from 0 to 3 (Absence of injury, Mild injury, Moderate injury, and Severe injury). Parameters of injury included tubular dilation, cellular necrosis, tubular casts, cellular vacuolization, inflammation, and congestion (33).

***Measurement of Bax and Bcl2***

The ELISA test was used to measure the levels of Bax and Bcl2 according to the kit instructions (ZellBio, Germany), and the values were expressed as ng/ml (34).

***Statistical analyses***

Two-way ANOVA and then *post-hoc* Tukey’s tests were used to compare the quantitative variables between the groups, in case of meeting the assumptions (normal distribution of data); otherwise, the Kruskal-Wallis test was used. The data were statistically analyzed using GraphPad Prism 8. The significance level was considered at 0.05

## Results

***Effects of TR and CR diets on the functional renal parameters before and after AKI***

There was no difference between the groups in the functional renal parameters (GFR, serum urea and creatinine, albumin excretion rate, and urea creatinine ratio) before AKI induction. 

After AKI, serum urea and creatinine, albumin excretion rate, and urea creatinine ratio increased (*P*<0.001) and GFR decreased in all groups compared with before AKI (*P*<0.001). 

After AKI, CR and TR diets prevented the increment of serum urea, so the amount of this index in both of the two groups was lower than sham CTL (*P*<0.001 and *P*<0.01, respectively). However, the effect of CR diet was more than TR (*P*<0.01). Also, this index decreased in the OVX CR group compared with OVX CTL (*P*<0.001) and the urea level in the OVX CR group was lower than OVX TR and Sham CR groups (*P*<0.001). Furthermore, this index decreased in OVX TR compared with Sham TR (*P*<0.001).

After AKI, serum creatinine decreased in sham TR, CR, and OVX CR groups compared with sham CTL and OVX CTL groups, respectively (*P*<0.001). In addition, the effect of CR diet in sham was higher than TR diet (*P*<0.01). Also this index level in OVX CR was lower than OVX TR (*P*<0.05) and Sham CR (*P*<0.01) groups. Furthermore, serum creatinine decreased in OVX TR in comparison with Sham TR (*P*<0.01). 

In the sham group only CR diet could decrease albumin excretion rate (*P*<0.001), but both of TR and CR diets decreased this indicator in the OVX rats (*P*<0.001 and *P*<0.05, respectively). Although, this indicator in OVX CR was lower than in OVX TR group (*P*<0.05). After AKI, both TR and CR diets increased GFR in the sham TR and CR groups comparison with sham CTL (*P*<0.05). Also, this indicator decreased in the OVX CR (*P*<0.001) and OVX TR (*P*<0.05) groups compared with OVX CTL (Tables 1 and 2).

***Effects of TR and CR diets on the kidney and BW before and after AKI***

Before AKI, BW reduced in both sham CR (*P*<0.001) and sham TR (*P*<0.05) groups compared with CTL. However, weight loss in the sham CR group was more than sham TR (*P*<0.001). BW in the OVX CTL group increased compared with sham CTL (*P*<0.001), and decreased in OVX CR compared with OVX CTL (*P*<0.001). Moreover, BW in CR diet in OVX was lower than OVX TR (*P*<0.01) and Sham CR (*P*<0.001). This index in OVX TR decreased in comparison with Sham TR (*P*<0.001). However, after AKI, only CR diet both in sham and OVX reduced BW (*P*<0.001 and *P*<0.05, respectively) (Figure 3A).

Before AKI, KW in the OVX group for both diets was lower than in similar sham groups (*P*<0.05). 

But after AKI, KW increased so this index in sham and OVX CTL groups was more than their control groups before AKI (*P*<0.001). After AKI, KW in sham CR was lower than sham CTL (*P*<0.001), also this index decreased in OVX CR in comparison with Sham CR (*P*<0.05) (Figure 3B). There was no difference between the groups in kidney weight/body weight (KW/BW) ratio, but after AKI, KW/BW ratio increased in sham and OVX CTL groups compared with their CTL groups (*P*<0.001). However, none of the diets prevented this index from rising (Figure 3C). 

***Effects of different diets on Bax BCL2 levels in kidney tissue before and after AKI***

Before the injury, none of the TR and CR diets were able to affect the kidney levels of Bax and Bcl2, but after AKI, Bax kidney level increased in all groups compared with similar groups before AKI. The Bax level in both sham and OVX, TR and CR groups were lower than their CTL (*P*<0.01) (Figure 4A). Compared with Bax, Bcl2 kidney level decreased after AKI, in addition, this index in TR and CR groups was higher than in their CTL groups (*P*<0.001). Also Bcl2 levels in OVX CR and OVX TR were lower than in Sham CR (*P*<0.05) and Sham TR (*P*<0.01) (Figure 4B). 

Although the Bax/Bcl2 ratio in all groups was similar before AKI, this indicator increased in both sham and OVX after AKI, so this Bax/Bcl2 ratio in sham and OVX CTL groups was higher than their CTL before AKI (*P*<0.001). After AKI, this index in sham TR and sham CR groups was lower than sham CTL) *P*<0.001). Similar results were observed in OVX rats, and this ratio in CR (*P*<0.001) and TR (*P*<0.01 (groups was lower than OVX CTL (Figure 4C). 

***Effects of TR and CR diets on damage/histopathological data of kidney tissue before and after AKI***

In all groups after AKI, damage/histopathological data (cell vacuolization, tubular dilatation, intratubular cast, cellular necrosis, inflammation, and congestion) increased compared with the groups before AKI. However, cell vacuolization in sham and OVX groups increased compared with similar groups before AKI (*P*<0.001), but only CR could prevent an increase in this index compared with sham and OVX CTL (*P*<0.05) (Figure 5A). In addition, tubular dilation was higher in all groups after AKI compared with similar groups before AKI (*P*<0.001), while both diets prevented an increase in this index, so the amount of tubular dilation in TR and CR groups was lower than in sham CTL and OVX CTL groups (*P*<0.05) (Figure 5B). Changes in intratubular cast was similar to cell vacuolization changes so that only CR prevented an increase in this index and there was a significant difference between this group with sham CTL and OVX CTL(*P*<0.05) (Figure 5C). Likewise, cellular necrosis increased in sham CTL and OVX CTL groups after AKI compared with before AKI (*P*<0.001). TR and CR diets decreased this index in sham TR and CR groups compared with sham CTL (*P*<0.05). but only CR was able to decrease this indicator in OVX CR compared with OVX CTL (*P*<0.05) (Figure 5D). The changes in inflammation and congestion were similar, after AKI, inflammation and congestion increased in sham and OVX CTL groups compared with before AKI (*P*<0.001). TR and CR diets in sham (*P*<0.01 and *P*<0.05, respectively) and only CR in OVX (*P*<0.05) prevented an increase in this index compared with sham CTL and OVX CTL groups, respectively (Figures 5E and 5F).

**Figure 2:**
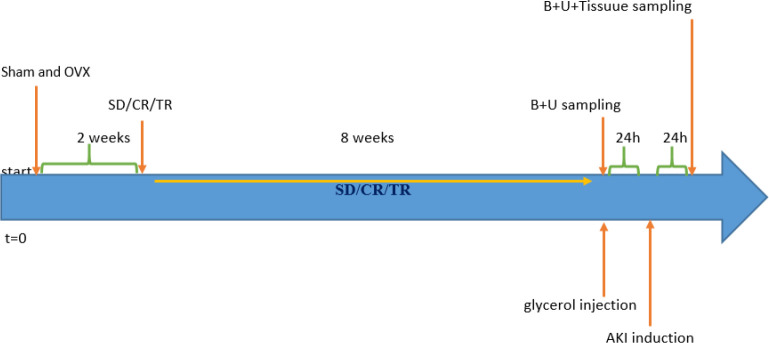
Schematic diagram of the representation of the experimental protocol. B: blood, U: urine, T: tissue, SD: standard diet, TR: time restriction diet, CR: calorie restriction diet, OVX: ovariectomized, Sham: ovary-intact, and CTL: control

**Figure 3:**
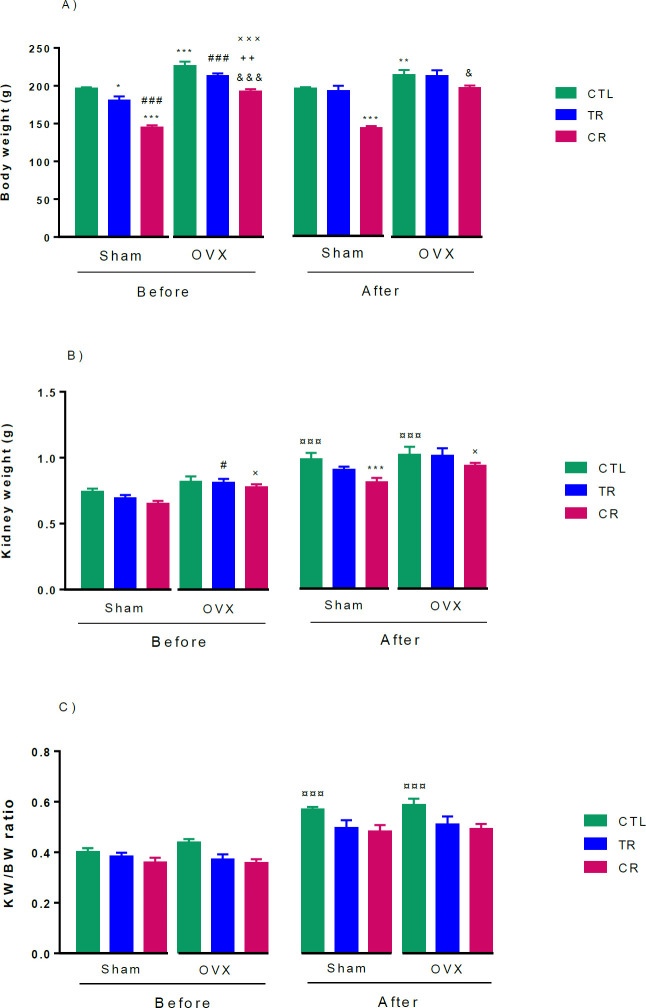
Effects of TR and CR diets in sham and OVX rats: A) BW (g), B) KW (g), and C) KW/BW ratio. Results are expressed as mean ± SEM, n = 6. ¤ *P*<0.05 and ¤¤¤ *P*<0.001, vs similar groups before AKI. * *P*<0.05, ** *P*<0.01, and *** *P*<0.001, vs Sham CTL. # *P*<0.05 and ### *P*<0.001, vs Sham TR. & *P*<0.05 and &&& *P*<0.001, vs OVX CTL. × *P*<0.05 and ××× *P*<0.001, vs Sham CR. OVX: ovariectomized, Sham: ovary-intact, CTL: control, TR: time restriction, CR: caloric restriction

**Table 1:** The effects of TR and CR diets on renal parameters in Sham and OVX rats before AKI

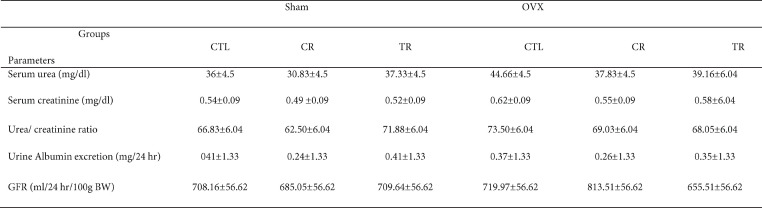

CR: caloric restriction, TR: time restriction, Sham: ovary-intact, OVX: ovariectomized, AKI: acute kidney injury

**Table 2:** The effects of TR and CR diets on renal parameters in Sham and OVX rats after AKI

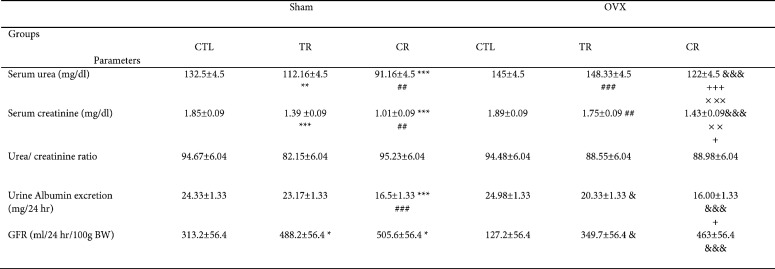

Data are expressed as mean ± SEM, n=6 rats/group. B: Before AKI, A: After AKI, * *P*<0.05, ** *P*<0.01, and *** *P*<0.001 vs Sham CTL. & *P*<0.05, && *P*<0.01, and &&& *P*<0.001, vs OVX CTL. ## *P*<0.01 and ### *P*<0.001, vs Sham TR. + *P*<0.05 and +++ *P*<0.001, vs OVX TR. ×× *P*<0.01 and ××× *P*<0.001, vs Sham CR. CTL: control, CR: caloric restriction, TR: time restriction, Sham: ovary-intact, OVX: ovariectomized, AKI: acute kidney injury

**Figure 4:**
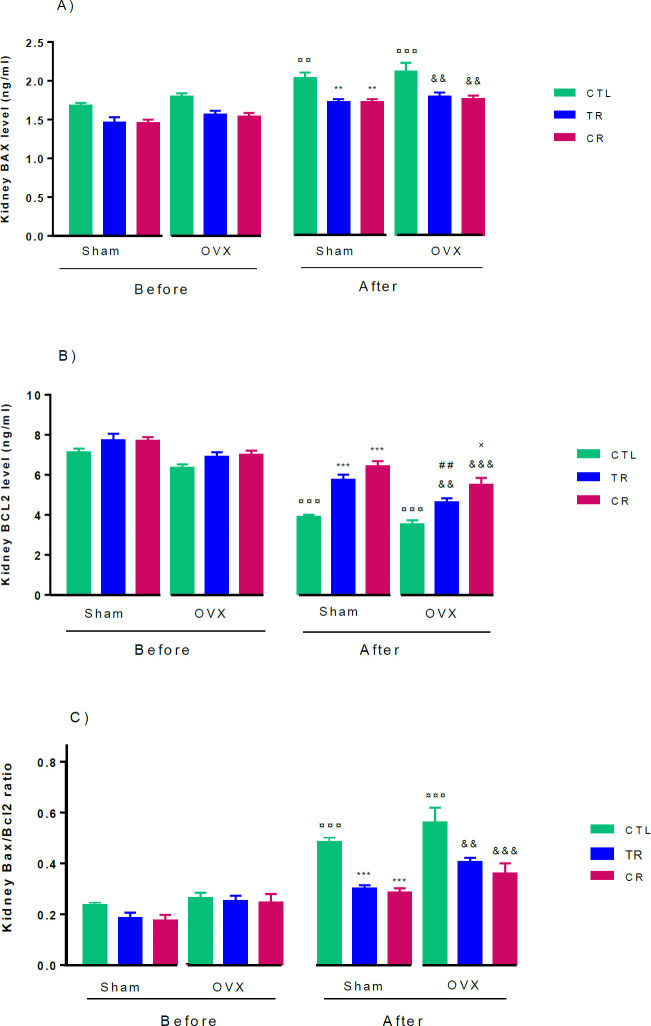
Effects of TR and CR diets in sham and OVX rats: A) Bax (ng/ml), B) Bcl2 (ng/ml) levels, and C) Bax/Bcl2 ratio. The results are expressed as mean ± SEM, n = 6. ¤¤¤ *P*<0.001 and ¤¤ *P*<0.01 vs similar groups before AKI. ** *P*<0.01 and *** *P*<0.001, vs Sham CTL. && *P*<0.01 and &&& *P*<0.001, vs OVX CTL. × *P*<0.05, vs Sham CR. ## *P*<0.005, vs Sham TR. OVX: ovariectomized, Sham: ovary-intact, CTL: control, TR: time restriction, CR: calorie restriction

**Figure 5:**
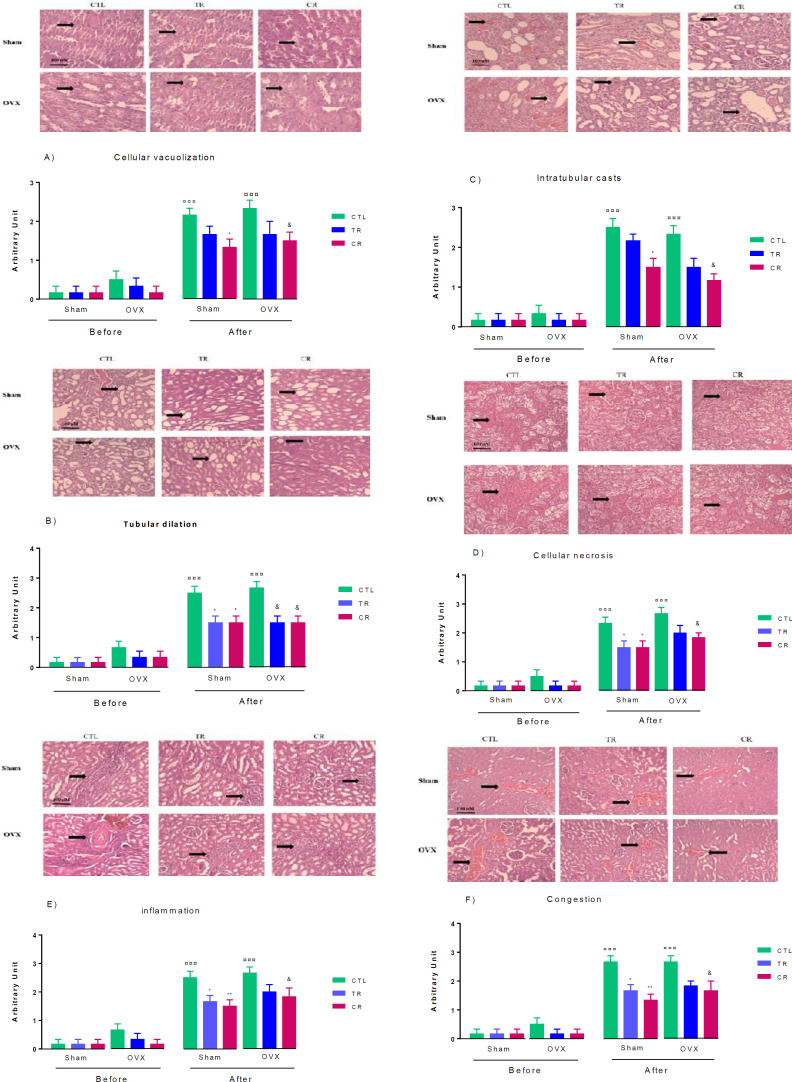
The effect of TR and CR diets on histopathological changes in sham and OVX rats: A) Cellular vacuolization, H&E X400, B) Tubular dilation, H&E X200, C) Intratubular casts, H&E X200, D) Cellular necrosis, H&E X200, E) Inflammation, H&E X200, F) Congestion levels. H&E X200. Results are expressed as mean ± SEM, n = 6. ¤ *P*<0.001, vs similar groups before AKI. * *P*<0.05 and ** *P*<0.01, vs Sham CTL. & *P*<0.05 and && *P*<0.01 VS OVX CTL. CTL: control, CR: caloric restriction, TR: time restriction, Sham: ovary-intact, OVX: ovariectomized

## Discussion

In the present study, the renoprotective effects of TR and CR diets on AKI were evaluated with emphasis on the biochemical, histopathological changes, and apoptosis in OVX animals (menopause model). These diets exerted different effects on the severity of symptoms before and after experimental AKI. The main ﬁndings of this study included 1) Both TR and CR diets increased GFR in OVX and ovary-intact animals after AKI; moreover, the CR diet reduced serum urea and creatinine and urinary albumin excretion in both groups, but the TR diet only reduced serum urea and creatinine and in ovary-intact animals. 2) Before and after AKI, none of the diets changed KW/BW ratio in the OVX and ovary-intact rats. 3) After AKI, Bax/Bcl2 ratio increased in all groups compared with before AKI, although both diets prevented this increment, indicating that TR and CR diets inhibited apoptosis. 4) Histopathological changes were observed in AKI rats, but these indicators showed better renal health status among two diets of TR and CR in OVX and ovary-intact rats.

After AKI, TR and CR diets prevented an increase in serum urea and creatinine as well as urinary albumin excretion and a decrease in GFR. Results of a study have shown that different models of dietary restriction are effective in protecting the kidney against AKI (35). It was also found that progression of kidney disease can be delayed by caloric restriction by decreasing the levels of urine protein, creatinine, and urea nitrogen and enhancing rodent models’ survival rate by reducing disease onset (36).

The possible mechanisms of positive effects of TR and CR diets in reducing kidney injury include improving mitochondrial function (37), affecting various transcription factors (38), increasing expression of SIRTs (39), decreasing the production of mitochondrial superoxide (40), reducing muscle mass, and increasing urinary creatinine excretion (41). 

In the other part of this study, glycerol injection led to enlarged kidneys in AKI animals, the KW/BW ratio increased, indicating injury after AKI. Based on the results, glycerol treatment also resulted in the development of renal edema, indicating an increase in kidney KW/BW ratio (42). None of the diets affected this ratio. In line with the current study, it has been found that injection of glycerol into the muscle leads to rhabdomyolysis and release of potentially toxic intracellular components, such as myoglobin, into the systemic circulation. It ultimately leads to the accumulation of myoglobin and iron in the kidney, intraluminal cast formation, and swelling of the kidneys, resulting in an increase in KW/BW (43). 

Histopathological results in AKI rats indicate that indicators of injury (inflammation, cellular necrosis, cellular vacuolization, tubular dilation, intracellular casts, and congestion) decreased in TR and CR diets, but effect of the CR diet was more than TR. Consistent with the current study results, histopathological studies have indicated significant structural changes, including necrosis, vacuolation, tubular dilatation, and cellular debris in the kidneys of glycerol-treated rats (44). It has been shown that CR animals have low blood lymphocyte levels, reducing inﬂammatory cytokines production by white blood cells in response to stimulation (45). Moreover, recent data have suggested that CR also has a strong anti-inflammatory effect on humans (46). Several metabolic and neuroendocrine mechanisms are responsible for this CR-mediated anti-inﬂammatory effect, including increased cortisol (47) and ghrelin production (48), reduced plasma glucose and advanced glycation end-product concentrations, low adiposity (49), reduced secretion of proinﬂammatory adipokines and cytokines (50), and increased parasympathetic tone (51). The results also indicated that CR is effective in reducing renal necrosis. CR has been shown to be involved in protecting cisplatin-induced renal injury and reducing acute tubular necrosis by increasing mRNA and protein levels of peroxisome proliferator-activated receptor-alpha target genes, which is in line with the present study (52). In the present study, it has also been indicated that CR diet decreased vacuolation and both of the diets reduced tubular dilatation after AKI. It has been reported that CR reduces vacuolation and tubular dilatation during AKI, maintains kidney tissue integrity, and improves renal situation and function (53), which is consistent with the present study. It has also been shown that fasting before or after cisplatin treatment improve the histopathological results of cisplatin-induced nephrotoxicity (53). Another study found that TR and CR diets reduce atrophy and dilate renal tubules in diabetic animals (54) and can improve histological parameters (55). 

In another part of this study, after AKI, CR and TR diets were able to decrease Bax levels and increase Bcl2 levels and improve Bax/Bcl2 ratio. The Bcl-2 family has been known to play an important suppressive role in regulating apoptosis (56). Increased Bax level and decreased Bcl-2 level have been observed in older kidneys (21). It has been shown that CR decreased the Bax/Bcl-2 ratio, likely through a lower activation of the intrinsic apoptotic pathway, which is consistent with the current study (52). Ischemic apoptosis in the kidney is a major cause of injury, so inhibition of the apoptotic process prevents inflammation and kidney injury (37). It has been reported that increasing Bax expression and Bax/Bcl-2 ratio, decreasing Bcl-2, which are essential components of the apoptosis cascade process (57), and CR with its antioxidant function significantly modulated the apoptosis process components and reduced apoptosis (21). The findings indicated that the anti-apoptotic function of CR was by suppressing Bax expression and activating caspase-3 (58). On the other hand, the TR diet had protective effects on the heart by reducing apoptosis and preserving cells (59). Another study reported that preoperative fasting had a protective role against renal IRI by inhibiting apoptosis (60). 

## Conclusion

In the present study, it was found that TR and CR diets are effective in reducing kidney injury during AKI, and estrogen deficiency has an effect on the severity of kidney injury. Histopathological results confirmed the renoprotective effects of TR and CR diets. Both TR and CR diets reduced the Bax/Bcl2 ratio and probably improved apoptosis. This study emphasized the protective role of pre-injury diets in the pathophysiological conditions of the kidney. Further studies are suggested to evaluate the beneficial effects of these diets in the presence of sex hormones.

## Authors’ Contributions

FDM, MK, MI, ZS, ARA, and ZH Conceived the study, performed data analysis and draft manuscript preparation, critically revised the paper, supervised the research, and approved the final version to be published.

## Conflicts of Interest

The authors declare that there are no conflicts of interest.
